# Epidemiologic characteristics of orthopedic surgical site infections and under-reporting estimation of registries using capture-recapture analysis

**DOI:** 10.1186/s12879-020-05687-z

**Published:** 2021-01-04

**Authors:** Niloufar Taherpour, Yadollah Mehrabi, Arash Seifi, Babak Eshrati, Seyed Saeed Hashemi Nazari

**Affiliations:** 1 Department of Epidemiology, School of Public Health and Safety, Shahid Beheshti University of Medical Sciences, Tehran, Iran; 2 Department of Infectious Diseases, Faculty of Medicine, Tehran University of Medical Sciences, Tehran, Iran; 3 Center for Preventive Medicine, Department of Social Medicine, Iran University of Medical Sciences, Tehran, Iran; 4 Prevention of Cardiovascular Disease Research Center, Department of Epidemiology, School of Public Health and Safety, Shahid Beheshti University of Medical Sciences, Tehran, Iran; 5 School of Public Health and Safety, Shahid Beheshti University of Medical Sciences, Daneshjoo Blvd, Evin Ave, Tehran, Postal code: 198353-5511 Iran

**Keywords:** Surgical site infection, Orthopedic surgery, Surveillance system, Capture-recapture

## Abstract

**Background:**

Surgical Site Infections (SSIs) are among the leading causes of the postoperative complications. This study aimed at investigating the epidemiologic characteristics of orthopedic SSIs and estimating the under-reporting of registries using the capture-recapture method.

**Methods:**

This study, which was a registry-based, cross-sectional one, was conducted in six educational hospitals in Tehran during a one-year period, from March, 2017 to March, 2018. The data were collected from two hospital registries (National Nosocomial Infection Surveillance System (NNIS) and Health Information Management database (HIM)). First, all orthopedic SSIs registered in these sources were used to perform capture-recapture (*N* = 503). Second, 202 samples were randomly selected to assess patients` characteristics.

**Results:**

Totally, 76.24% of SSIs were detected post-discharge. *Staphylococcus aureus* (11.38%) was the most frequently detected bacterium in orthopedic SSIs. The median time between the detection of a SSI and the discharge was 17 days. The results of a study done on 503 SSIs showed that the coverage of NNIS and HIM was 59.95 and 65.17%, respectively. After capture-recapture estimation, it was found that about 221 of orthopedic SSIs were not detected by two sources among six hospitals and the real number of SSIs were estimated to be 623 ± 36.58 (95% CI, 552–695) and under-reporting percentage was 63.32%.

**Conclusion:**

To recognize the trends of SSIs mortality and morbidity in national level, it is significant to have access to a registry with minimum underestimated data. Therefore, according to the weak coverage of NNIS and HIM among Iranian hospitals, a plan for promoting the national Infection Prevention and Control (IPC) programs and providing updated protocols is recommended.

## Background

Surgical Site Infection (SSI) is one of the most common surgical-related problems in the world, especially in developing countries [1]. SSI is a kind of Nosocomial Infections (NIs) - also called Health care-associated Infections (HAIs) that occur within 30 days of the procedure or in a one-year period if mechanical or prosthetic material is implanted at surgery [2]. SSI is responsible for mortality, long hospitalization period, and a high economic burden [3]. According to the past reports, the incidence rate of SSI is globally about 10–20% [4] and is the most frequent type of HAIs in low and middle income countries [5]. The most commonly reported microorganism is gram-negative *Escherichia-coli*, accounting for 6.7–50% of incidence, and the second one is gram-positive *Staphylococcus aureus*, causing SSI in procedures [6]. According to the European Center for Disease prevention and Control (ECDC), the percentage of SSI varies from 0.5 to 9.0%, depending on the type of procedure [7]. As stated by the World Health Organization (WHO), due to the limited and low quality of data in low and middle-income countries, the incidence and prevalence of SSIs are underestimated. Considering the reports of different countries, WHO estimated that the prevalence of HAI varies between 5.7 and 19.1% in low- and middle-income countries [8]. In Eastern Mediterranean regions, the overall prevalence of SSI was reported to be about 7.9% in 2019 [9]. The overall prevalence of HAI in Iran, as a middle-upper-income country, was about 4.5% in 2017, where bloodstream infections, surgical site infections, and pneumonia were the most common types of HAIs in Iran, respectively [4].

Due to the nature of orthopedic surgeries and special patients under this procedure with variety of conditions and disorders (older patients especially in arthroplasty surgeries, underlying diseases and penetrating trauma) the risk of surgical site infection is higher compared to other procedures. The incidence of orthopedic SSIs in Iran was 8.8% in 2018; This type of infection has been responsible for long hospitalization period and bad prognosis [10, 11].

In Iran, there are two hospital information sources for registering HAIs. One of these information sources is Health Information Management (HIM) that plays a role in maintaining and collecting the medical records. In this unit, all medical and health information of patients is registered based on ICD-10 and ICD-9-CM coding system [12]. The second source is the National Nosocomial Infections Surveillance system (NNIS), a computer-based software, which was launched in 2011. In this Iranian surveillance system, the information of HAIs such as demographic characteristics of patients, vital status of them, Length of stay (LOS) or the duration of hospitalization, type of HAIs, device utility days, laboratory information by hospitals and wards are recorded [13]. Therefore, since these two sources of information do not depend on one another, some HAIs may not be detected by the NNIS. Also, owing to the lack of up-to-date instructions and the occurrence of human errors, the diagnosed HAIs may not be reported completely, which results in underestimation.

Thus, to realize the real distribution and trends of diseases in order for controlling and preventing its outcomes in the country, complete data with minimum underestimation is required. This study was conducted aiming at the estimation of the number of orthopedic SSIs from 2017 to 2018 using the capture-recapture method. At the second stage, the existing problems in NNIS among hospitals under study in Iran were reported.

## Methods

**Fig. 1:**
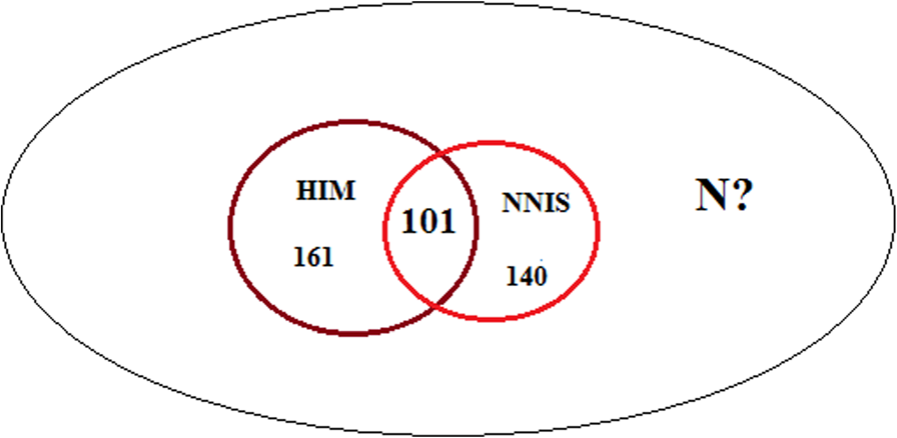
Distribution of orthopedic SSIs among six educational hospitals in Tehran based on two data sources

This registry-based, cross-sectional study was conducted in six educational hospitals in Tehran from March, 2017 to March, 2018. First, six hospitals (over 250 beds) were randomly selected. After that, data were collected from two sources in hospitals. One of them is the information of patients infected in orthopedic surgeries from 21 March, 2017 to 21 March, 2018 - orthopedic SSIs were based on the health information management (HIM) reporting. The second one was the orthopedic SSIs which were based on National Nosocomial Infections Surveillance system (NNIS) registries in each six mentioned hospitals. Overall, we collected 262 and 241 identical codes of SSIs from HIM and NNIS, respectively from 2017 to 2018 (*N* = 503, after the exclusion of duplicates) Fig. 1. Five hundred-three SSIs were used for estimating the orthopedic SSIs population size using two- sources capture-recapture method. After that, from 503 identical codes, 202 medical records of orthopedic SSIs were randomly selected in order for monitoring the orthopedic SSI patients` characteristics.

### Two-source capture-recapture

In ecologic and epidemiologic studies, capture-recapture sampling is a method used to estimate the completeness of ascertainment of disease registers and estimate the unknown size of a population [14]. We used a less biased alternative estimator of population size which is given by the Chapman estimator [15, 16]. Also, categorical data (as frequencies and percentages) and quantitative data (median and interquartile range (IQR)) were calculated. To describe and compare subjects, T-test, Mann-Whitney and Chi-square (χ2) tests at significant level of < 0.05 were used. Data analysis was carried out using STATA.14 software.

## Results

The results of studying 503 SSIs in six educational hospitals during the 2017–2018 period showed that the coverage percentages based on existing data in NNIS and HIM were 59.95 and 65.17%, respectively (Table 1).

**Table 1:** Results of the number of SSIs reported by NNIS compared to HIM among six educational hospitals under study in Tehran from March 2017 to March 2018

Hospital code	(a) HIM reported cases (n)	(b) NNIS reported cases (n)	(c) Common cases between two registries ***n*** (c/d%)	(d) Total number of cases (***n***) (a + b-c)	(e) Coverage of NNIS (b/d %)	(F) Coverage of HIM (a/d %)
Hospital 1	24	28	6 (13.04)	46	60.86	52.17
Hospital 2	6	6	0 (0)	12	50	50
Hospital 3	60	45	14 (15.38)	91	49.45	65.93
Hospital 4	42	6	2 (4.34)	46	13.04	91.30
Hospital 5	75	66	34 (31.77)	107	61.68	70.09
Hospital 6	55	90	45 (45)	100	90	55
Total	262	241	101 (25.12)	402	59.95	65.17

According to the results obtained from capture-recapture estimation, about 221 of SSIs were not detected by two sources in six hospitals and the real number of SSIs were estimated to be 623 ± 36.58 (95% CI, 552–695). Furthermore, the completeness percentages of HIM and NNIS based on the capture-recapture method were 42.05 and 38.68%, respectively and under-reporting was 63.32%. This indicates the completeness of SSI registries was low, and about half of the cases were missed among the respective six hospitals in Tehran from 2017 to 2018.

In Table 2, among 202 orthopedic SSIs in the six respective educational hospitals, 148 (73.26%) were males and 54 (26.73%) were females. The median age of orthopedic SSI cases was 44 (32–62); 48 (23.76%) SSIs were identified pre-discharge and 154 (76.24%) of them were detected post-discharge. Overall, 92 (45.55%) SSIs occurred during Open Reduction and Internal Fixation (ORIF), 80 (39.60%) in other orthopedic surgeries, 19 (9.40%) in Total Knee Arthroplasty (TKA), and 11(5.45%) Total Hip Arthroplasty (THA). *Staphylococcus aureus* (11.38%) and *Klebsiella* (10.89%) were the most frequent bacteria in orthopedic SSIs. Furthermore, 5 (2.47%) cases died due to orthopedic SSIs. A statistically significant association was observed between the type of microorganism (*P* = 0.001) and time between surgery to infection (*P* < 0.001) with occurrence of SSIs (post or pre discharge).

**Table 2:** Characteristics of SSIs in orthopedic surgery in six educational hospitals under study in Tehran from March 2017 to March 2018

Variable	Post-discharge infection	Pre-discharge infection	Total	***P***_value
**Sex**				0.152
Female	45 (83.33)	9 (16.67)	54	
Male	109 (73.65)	39 (26.35)	148	
**Vital status**				0.388
live	151 (76.65)	46 (23.35)	197	
Dead	3 (60.0)	2 (40.0)	5	
**Type of procedure**				0.218
ORIF	73 (79.35)	19 (20.56)	92	
TKA	16 (84.21)	3 (15.79)	19	
THA	9 (81.82)	2 (18.18)	11	
Other orthopedic surgeries	56 (70.0)	24 (30.0)	80	
**Bacteriology test**				0.001^*^
*E. coli*	6 (75.0)	2 (25.0)	8	
*Entrobacter*	6 (37.50)	10 (62.50)	16	
*Klebsiella*	12 (54.55)	10 (45.45)	22	
*Pseudomonas aeruginosa*	6 (85.71)	1 (14.29)	7	
*Staph aureous*	18 (78.26)	5 (21.74)	23	
*Staph epidermis*	7 (87.50)	1 (12.50)	8	
*Acinetobacter*	3 (60.0)	2 (40)	5	
Others^1^	9 (90.0)	1 (10.0)	10	
Unknown	87 (76.24)	16 (15.53)	103	
**Age (years)**	46 (33.75–62.25)	38.5 (28–55.75)	44 (32–62)	0.076
**Time between surgery to infection (days)**	36.5 (20.5–85.5)	9 (5.25–16)	27.5 (14–60)	< 0.001^*^
**Length of hospitalization due to orthopedic surgery (days)**^**2**^	10 (6–17.25)	13 (8–23)	11 (7–19)	0.052
**Duration of hospitalization due to infection (days)** ^**3**^	17 (8–28)	17 (7.25–30)	17 (18–29)	0.685
**Total**	154 (76.24)	48 (23.76)	202 (100)	–

Data are *n* (%) or median (Q1-Q3).

^1^ Such as *Cocci*, *Proteous, Candida*, *Streptococ viridans* and *Entrococus Faecium*

^2^ Time between admission to discharge among the post-discharge infection group and duration between admission to detection of SSI among the pre-discharge infection cases

^3^ The duration between detection of SSI to discharge

The median length of hospitalization due to surgery before infection occurrence was 11 (7-19) days. After operation, SSIs led to longer hospitalization with median of 17 (8-29) days. Also, the median length of surgery to infection occurrence was 27.5 days, which has been longer among post discharge SSIs (36.5 days) (*P* < 0.001) (Table 2).

According to the Table 3, 110 (54.46%) orthopedic SSIs were totally detected within 30 days after the procedure. Furthermore, 17.82% of them were identified within 90 days to one year after the procedure. The details of SSIs characteristics are listed in Table 3. (Table 3).

**Table 3:** Relative frequency of infection occurrence in different intervals after orthopedic surgery from March 2017 to March 2018

Type of procedure (ICD9_CM)	≤ 30 (days) ***n*** (%)	31–60 days ***n*** (%)	61–90 days ***n*** (%)	> 90 days ***n*** (%)	Total
**ORIF** ^**1**^	48 (52.17)	20 (21.74)	6 (6.52)	18 (19.57)	92
**TKA** ^**2**^	13 (68.42)	3 (15.79)	1 (5.26)	2 (10.53)	19
**THA** ^**3**^	6 (54.55)	2 (18.18)	2 (18.18)	1 (9.09)	11
**Other orthopedic procedures** ^**4**^	43 (53.75)	17 (21.25)	5 (6.25)	15 (18.75)	80
**Total**	110 (54.46)	42 (20.79)	14 (6.93)	36 (17.82)	202 (100)

^1^ Open Reduction and Internal Fixation

^2^ Total knee arthroplasty

^3^ Total hip arthroplasty

^4^ Such as Closed Reduction and Internal Fixation, amputation, correction of deformity, and ilizarov

## Discussion

In the present study, the median age of SSIs was about 44 and most of them were males (73.26%). About 2.47% of cases died due to orthopedic surgical site infection. These results are in line with other literatures in which SSI was more among males and middle-aged patients with fatality rate of 2–3%. However, Al-Mulhim, et al. found that SSI was more common in younger patients with an average age of 38 in Saudi Arabia [17, 18].

Surgical site infection may lead to many complications, among which one is prolonged hospitalization due to infection. In this study, the median days of hospitalization due to orthopedic surgery was 17 days while the median length of hospitalization due to SSI was 11 days. This finding is similar to other prior studies that reported the mean of hospitalization days due to SSI; it varied from 4 to 32 days and the average length of increased hospitalization due to orthopedic SSIs was 19.8 days while the average length of stay for patients with no post-surgical infection was lower at 9.1 days [19–21]. Thus, SSI can increase the economic and psychological burden on patients. It is Important to recognize the risk of being infected among patients using risk assessment and to follow HAI prevention protocols in surveillance system. In this study, a statistically significant association was found between the type of microorganisms with the occurrence of SSIs in post- discharge or pre-discharge time. In addition, the average time interval between surgery to the incidence of infection has been significantly longer among post-discharge SSIs; this could occur due to different incubation and infectious periods among a variety of microorganisms and the effect on the appearance of infection symptoms after surgery [22]. However, the length of time between infection occurrence and its detection may be delayed because of the immune system response, consumption of prophylaxis, and being unaware of the infection symptoms.

After study on post- discharge and pre-discharge SSIs, it was observed that 76.24% of SSIs occurred after discharge, among which 45.54% occurred within 31 days after orthopedic procedure and, totally, 17.82% of SSIs were detected within 90 days to one year after orthopedic surgery. This varies by the type of orthopedic procedures. So that, 19.57% of SSIs due to ORIF procedures occurred post-discharge within 90 days to one year after surgery. These results were in line with other studies in which it was reported that the length of time between discharges to the detection of orthopedic SSIs varied between 8 days to 8 months. In another study, it was reported that most SSIs were detected after the 21st postoperative day [23, 24]. As a result, this fact shows the importance of post-discharge surveillance, especially in procedures in which mechanical or prosthetic materials are implanted during surgery. Therefore, SSIs can occur in a late phase and post-discharge. So, performing a post-discharge surveillance can help the timely detection of SSIs and can prevent missing cases and under-estimation in registries systems.

As it was mentioned, In Iran, there are two independent registries in each hospital. One of them is HIM that generally recognizes and registers all disease cases using ICD-10 and ICD-9 criteria from medical reports of each patient. The other is NNIS that registers HAIs in each hospital. After collecting all reported HAIs from hospitals in the country, based on the NNIS report, all of them are reported to Iranian Center for Communicable Disease Control (ICCDC) in the Ministry of Health and Medical Education [25]. Thus, the number of HAIs and the incidence rate in country are based on case finding and case reporting of NNIS to upstream centers. After evaluation of the mentioned registry performance among six educational hospitals from March, 2017 to March, 2018, it was found that among 402 SSIs, just 241 records were reported from NNIS with 59.96% of total coverage (ranges from 50 to 90%). About 161 orthopedic SSIs that were registered based on medical reports of patients were missed by NNIS. As a result, it can be said that the performance of NNIS among the mentioned hospitals was weak and about half of cases were not detected by NNIS. So, reported incidence rates are underestimated. According to a study done by Seifi, et al., which was conducted in a hospital in Iran, sensitivity, specificity, and positive and negative predictive values of routine surveillance were 27.5, 97.2, 69, and 85.3%, respectively [26]. Thus, we can say the performance of the routine surveillance system is poor. To have an effective case finding and increasing the data coverage, some changes are required to be made to the infrastructure and case finding protocols.

Furthermore, after capture-recapture was observed, the completeness of NNIS was 38.68%. In other words, under-reporting of orthopedic SSIs among six educational hospitals was about 63.32%. This finding was in line with another study in which under-reporting of SSIs in ICU was 82.2% in 2019 [26]. Under-reporting of SSIs in registry system is unavoidable; however, this issue can be overcome by recognizing the existing problems in the system. The possible problems in the NNIS system causing under-reporting are as follows:

1. Lack of data linkage between emergency units of hospitals and private clinics with NNIS:
  In Iran, only in-patients with severe HAIs, are registered as infected cases. Therefore, probable HAIS that are referred to private clinics or hospital emergency rooms are not being recorded as nosocomial infections and it leads to possibly of missing patients with subclinical infections;
2. Lack of data linkage between HIM unit and NNIS;
3. Lack of active surveillance system: NNIS in Iran is currently a passive surveillance, it means that health care workers report notifiable nosocomial cases on a case-by-case basis and it is impossible to ensure compliance by health care providers. so, it leads to under-reporting [27, 28];
4. Lack of post-discharge surveillance;
5. Non-reliable laboratory investigation methods (false-negative results): prescription of antibiotics, as a routine implementation before surgeries, can affect the results of laboratory tests [29] and can lead to false-negative and under-reporting;
6. Limited human and technical resources to register and quality control of NNIS.

With regard to the existing problems, it is recommended to plan for updating the existing prevention and controlling NNIS protocols in Iran. Some actions that can be taken are antibiotic prescription monitoring, assigning and retraining health care workers with experience of registry management and infection prevention and control, planning to provide the infrastructure for linkage of data among hospitals, clinics, and emergency units using electronic medical records, taking advantage of Standardized Infection Ratio (SIR) as a summary measure recommended by the National Healthcare Safety Network (NHSN) to track Health care-associated Infections (HAIs) [30], assigning a post-discharge surveillance according to the CDC ‘s recommendation based on monitoring the patients within 30 days of a surgical procedure or up to 90 days for implanted prosthetics [31]. Although this issue is difficult and challenging to implement, using active surveillance can be implemented and accessible. For example, using telephone interview as a diagnostic tool for post-discharge surveillance and follow-up of patients, have shown good results in terms of reliability and validity (72% sensitivity and 100% specificity), [32] using questionnaires reported by physicians or surgeons and health care workers in local health centers [33], and providing advice to patients at the time of discharge to return for post-operative visits [34] can help to prevent and decrease the missed HAIs.

Some limitations of the current study should be noted. First, because of the lack of time and the large number of hospitals, we could not conduct a study on all educational hospitals in Tehran province. Second, since private hospitals did not cooperate, we conducted this study in six educational hospitals in Tehran.

## Conclusion

In order for knowing the trends of mortality and morbidity in hospitals and in country also for aiding health care services for planning prevention strategy, it is important to have access to a registry with minimum underestimated data. Hence, according to the moderate coverage of NNIS and HIM data sources among Iranian hospitals, it is highly necessary to promote the national Infection Prevention and Control (IPC) programs and to provide updated protocols.

## Data Availability

An aggregated version of the data might be made available from the corresponding author on reasonable request with permission from the ethics committee.

## Abbreviations

ECDC: European Center for Disease prevention and Control
HAIs: Health care-associated infections
HIM: Health Information Management
IPC: Infection Prevention and Control
IQR: Interquartile Range
ICCDC: Iranian Center for Communicable Disease Control
LOS: Length of stay
NIs: Nosocomial Infections
NNIS: National Nosocomial Infection Surveillance System
NHSN: National Healthcare Safety Network
ORIF: Open Reduction and Internal Fixation
SSIs: Surgical Site Infections
SIR: Standardized Infection Ratio
THA: Total Hip Arthroplasty
TKA: Total Knee Arthroplasty
WHO: World Health Organization
